# Vitamin D Metabolites and the Gut Microbiome in Older Men

**DOI:** 10.1093/geroni/igaa057.3077

**Published:** 2020-12-16

**Authors:** Deborah Kado, Robert Thomas, Lingjing Jiang, John Adams, Rob Knight, Eric Orwoll

**Affiliations:** 1 University of California, San Diego, La Jolla, California, United States; 2 UCLA, Los Angeles, California, United States; 3 Oregon Health & Science University, Portland, Oregon, United States

## Abstract

We examined the bidirectional impact of vitamin D on the composition and diversity of the gut microbiome in 567 MrOS men. Vitamin D metabolites were measured using LC-MSMS and stool sub-operational taxonomic units defined from 16S ribosomal RNA sequencing data using Deblur and Greengenes 13.8. Men’s mean serum level of 25(OH)D was in the sufficient range. Faith’s Phylogenetic Diversity and non-redundant covariate analyses revealed that 1,25(OH)2D explained 5% of variance in α-diversity; the other non-redundant covariates of site, race, recent antibiotic and antidepressant use explained another 6%. In β-diversity analyses using unweighted UniFrac, 1,25(OH)2D was the strongest factor assessed, explaining 2%. Random forest plot analyses identified 12 taxa, 6 in the phylum Firmicutes, positively associated with either 1,25(OH)2D and/or [1,25(OH)2D/25(OH)D] activation ratio. Higher levels of the active 1,25(OH)2D, but not 25(OH)D, were associated with butyrate producing bacteria. Men with favorable vitamin D activation profiles also had greater gut microbial diversity.

